# Drug-Coated Balloon for the Treatment of Femoropopliteal Tosaka Class III In-stent Restenosis Lesions

**DOI:** 10.3389/fsurg.2020.616414

**Published:** 2021-01-13

**Authors:** Bihui Zhang, Guochen Niu, Ziguang Yan, Yinghua Zou, Xiaoqiang Tong, Min Yang

**Affiliations:** Department of Interventional Radiology and Vascular Surgery, Peking University First Hospital, Beijing, China

**Keywords:** drug-coated balloon, femoropopliteal lesions, in-stent restenosis, Tosaka class, patency

## Abstract

**Background:** To date, there have been few studies examining the efficacy and safety of drug-coated balloon (DCB) angioplasty in the treatment of Tosaka class III in-stent restenosis (ISR) lesions in the clinical setting. Therefore, this study aimed to investigate the clinical efficacy and safety of DCBs in patients with Tosaka class III ISR femoropopliteal lesions.

**Methods:** This single-center study enrolled 28 femoropopliteal ISR Tosaka III patients who were treated by DCB angioplasty from September 2016 to September 2018. The patency, the freedom from target lesion revascularization (TLR) rate, clinical improvement, and safety endpoints were analyzed during a 14-month follow-up period.

**Results:** Out of the 28 patients, 32.1% presented with critical limb ischemia. The mean lesion length was 250.4 ± 93.9 mm. Technical success was achieved in all lesions (100%). Debulking devices were used in 50% of lesions, and bailout stents were performed in 3.6% of patients. Kaplan Meier estimates that the 14-month primary patency was 79.2% (95% CI 60.6–97.8%), whereas the freedom from TLR rate was 91.5% (95% CI 80.1–100%). Clinical symptoms improved by at least 1 Rutherford category in 82.1% of limbs. The ankle-brachial index (ABI) values improved from 0.51 ± 0.30 to 1.05 ± 0.22 at the final follow-up (*P* < 0.001). The rate of freedom from 30-day major adverse limb events (MALEs) was 100%. The mortality rate was 7.1%.

**Conclusion:** These results suggested that the use of DCBs is safe and effective in treating femoropopliteal Tosaka class III ISR lesions.

## Introduction

Peripheral artery disease is the third leading cause of atherosclerotic cardiovascular morbidity and affects more than 200 million people globally (1). Recent guidelines recommend endovascular treatment of symptomatic aortoiliac and femoropopliteal patients as the first-line therapy (2, 3). Stents have been used to treat femoropopliteal lesions for decades and have shown better patency than plain old balloon angioplasty (POBA) (4). However, the 12-month patency in lesions longer than 10 cm remains poor with 50–65% rates, thereby leading to in-stent restenosis (ISR) (5–7). Treatment of femoropopliteal ISR lesions, especially Tosaka class III lesions, is intractable (8, 9).

Drug-coated balloons (DCBs) is an emerging therapeutic method, and multiple randomized controlled trials have demonstrated its superiority compared to POBA in reducing restenosis, minimizing target lesion revascularization, and decreasing late lumen loss (10–15). However, the majority of lesions included in these trials were *de novo*. Other studies have evaluated the use of DCBs in treating ISR lesions; however, these latter reports did not focus on Tosaka class III lesions and demonstrated conflicting outcomes (16–23). Therefore, the efficacy and safety of DCBs in Tosaka III ISR femoropopliteal lesions remains to be elucidated.

This study aimed to investigate the effectiveness and safety of DCBs in Tosaka class III femoropopliteal ISR lesions in real-world scenerio.

## Materials and Methods

### Patient Population

The local institutional Human Investigations Committee approved this retrospective study. Patient informed consent was waived for this study. Patients with femoropopliteal Tosaka class III ISR lesions treated with DCB from September 2016 to September 2018 were enrolled in this single-center study. The inclusion criteria were as follows: (1) patients age 18 years or older; (2) patients diagnosed with Rutherford Classification category 1 or greater; (3) the presence of femoropopliteal Tosaka III ISR lesions; and (4) the lesions were treatable with the available Acotec Orchid DCB. Pregnant patients or patients who were planning on becoming pregnant were excluded.

DCB treated a total of 120 femoropopliteal artery disease patients in the study period. Among them, 28 patients were diagnosed with femoropopliteal Tosaka III ISR lesions. According to institutional standard protocols, the patient's baseline characteristics including age, gender, cardiovascular risk factors, and clinical status stratified by the Rutherford classification were collated in a local database. Pre-procedural physical examination, ankle-brachial index (ABI) measurements, and vascular duplex sonography were performed.

### Endovascular Procedures

Patients were treated by experienced vascular specialists in a catheter lab under local anesthesia and supplemented with intravenous sedation when required. All treatment procedures, including the use of DCB or additional devices, were determined at the vascular specialists' discretion. All patients were treated with Orchid DCB (Acotec, Beijing, China). The balloon was coated with 3.0 μg paclitaxel per mm^2^ and magnesium stearate as the excipient. During the procedure, 6–7 French sheaths were chosen for artery access. Intraluminal or subintimal crossings were performed on occlusions using a 0.035-/0.018-inch hydrophilic guidewire. Debulking devices, including Silverhawk/Turbohawk (Medtronic), Rotarex catheter (Straub Medical AG), or Angiojet (Boston Scientific), were employed at the discretion of the vascular specialists. At least one uncoated balloon was used for pre-dilation before each DCB. The diameter of the DCB was 0.5 or 1 mm larger than the pre-dilation uncoated balloon. Vessel diameter and lesion characteristics were visually estimated. Lesion length was measured by a radiopaque ruler placed under the patient's body on the examination bed. The degree of calcification was not evaluated due to the interference of the metal stents. The length of the DCB was sufficient to cover at least 1 cm distal and proximal to the lesion. If more than one DCB was used, the overlap was at least 1 cm. Implantation of self-expanding stents was allowed in flow-limiting dissection cases or where residual stenosis was >30%. Inflow and outflow lesions were often treated during the same intervention as determined by the operators.

### Antiplatelet and Anticoagulation Protocol

Before the procedure, all the patients were administered a dual antiplatelet therapy of aspirin 100 mg/day and clopidogrel 75 mg/day for at least 7 days or a loading dose consisting of 300 mg aspirin and 300 mg/day clopidogrel 6 h before the procedure. During the procedure, heparin (5,000 IU) was administered for anti-coagulation following the long sheath insertion. All patients were prescribed dual antiplatelet therapy (aspirin 100 mg/day and clopidogrel 75 mg/day) for at least 6 months after the procedure and changed over to one agent after that. For patients with cardiovascular comorbidities, the administration of antiplatelet therapy was at the discretion of the doctors. Statin was prescribed for patients with dyslipidemia.

### Follow-Up

All patients underwent a physical examination and ABI measurements to assess the results of treatment before discharge. Follow-up visits at 1, 3, 6, and 12 months and yearly after that were routinely scheduled at our hospital's outpatient department. During the follow-up consultations, symptoms inquiry, physical examination, duplex ultrasound scanning, and ABI measurements were conducted. Computed tomography angiography (CTA) was performed every year or when the ABI decreased more than 20%, or if the duplex ultrasound scanning showed significant restenosis (>50%), or if the patient complained of severe symptoms. Patients who did not return for follow-up were contacted by phone consultation every 6 months.

### Endpoints and Definitions

The main endpoint of the study was the 14-month primary patency rate. The secondary endpoints were as follows: the 14-month freedom from target lesion revascularization (TLR) rate; the technical success rate; the 14-month mortality rate; and the 30-day major adverse limb events (MALEs) rate including death, major amputation, and TLR. Tosaka class III lesions were defined as totally occluded ISR lesions (8). In-stent chronic total occlusion (ISCTO) was defined as Tosaka class III lesions with a history of more than 3 months. Procedural technical success was defined as <30% residual stenosis in the final angiogram. Patency was defined as <50% restenosis based on CTA/angiography or duplex ultrasound scanning, with a 2.4 cutoff value for the peak systolic velocity ratio (PSVR). Primary patency was defined as uninterrupted patency without procedures, performed on or at the margin of the treated segment. TLR was defined as any intervention for treating the restenosis or other complication of the culprit vessels. Complications included local infection, dissection, thromboembolism, fistulas, hematoma, acute occlusion, renal failure, stroke, and myocardial infarction.

### Statistical Analysis

Statistical analyses were performed using SPSS version 20 (SPSS, Chicago, IL, USA) software. Continuous data are shown as means ± standard deviation, and categorical data are presented as count and percentage. The normally-distributed continuous variables were compared using 2-sided Student's *t*-tests, and the non-normally-distributed continuous variables were compared using the Wilcoxon rank test. Mean values of the baseline and immediate postoperative or follow-up measurements were compared using a paired *t*-test. Categorical variables were compared using the 2-sided likelihood ratio chi-square test or Fisher exact test. Kaplan-Meier survival analysis with a log-rank test was used to estimate primary patency and freedom from TLR. A Cox regression analysis was used to identify independent predictors of restenosis and TLR. Variables associated with restenosis in the univariate analysis were entered into a multivariable model (*P* < 0.05). Outcomes were depicted as hazard ratio (HR) and 95% confidence interval (CI). Results were considered statistically significant when *P* < 0.05.

## Results

### Patient Characteristics

Demographic information for the patients is shown in Table 1. The patients' mean age was 69.3 ± 8.5 years, and 67.9% of patients were male. Cardiovascular risk factors were highly prevalent, including hypertension, dyslipidemia, and diabetes. Thirteen (46.4%) patients had a history of smoking. Most patients (92.9%) presented with severe claudication (60.8%) or critical limb ischemia (32.1%) at baseline. The mean pre-procedure ABI was 0.51 ± 0.30.

**Table 1:** Patient characteristics.

**Variables**	**Total (*n* = 28)**
Age, y	69.3 ± 8.5
Male	19 (67.9)
Hypertension	22 (78.6)
Dyslipidemia	16 (57.1)
Diabetes	19 (67.9)
Nicotine	13 (46.4)
Coronary artery disease	6 (21.4)
Ischemic stroke	4 (14.3)
**Rutherford classification**
1	1 (3.6)
2	1 (3.6)
3	17 (60.7)
4	3 (10.7)
5	4 (14.3)
6	2 (7.1)
ABI	0.51 ± 0.30

*Continuous data are presented as means ± standard deviation; categorical data are shown as n (%)*.

### Lesion and Procedural Characteristics

The average total target lesion length was 250.4 ± 93.9 mm (Table 2). The majority of lesions (78.6%) were longer than 150 mm. Approximately two-thirds (67.9%) of the lesions were in both the femoral and the popliteal arteries, while 32.1% of lesions were restricted to the femoral arteries alone. Stent fractures were observed in two patients (7.1%). Nearly 90% of the lesions were ISCTO. Technical success was obtained in all cases. Debulking devices were used in 14 of the 28 lesions (50.0%). Specifically, Silverhawk/Turbohawk was used in four patients, Angiojet was used in two patients, and Rotarex was used in eight patients. An average number of 1.71 ± 0.60 DCBs were used in each patient. A bailout stent was performed in only one patient (3.6%). Three complications (10.7%) occurred in the process of endovascular treatment, including 2 cases of thrombosis and 1 case of distal embolization. All conditions were managed via endovascular procedures, and open surgery was not required. The complications did not elongate the length of hospitalization. Within 30 days after the procedure, no MALEs had occurred. The rate of freedom from 30-day MALEs was 100%.

**Table 2:** Lesion and procedural characteristics.

**Variables**	**Total (*n* = 28)**
Lesion length (mm)	250.4 ± 93.9
Length > 150 mm	22 (78.6)
Femoral and popliteal artery lesions	19 (67.9)
Solitary femoral artery lesions	9 (32.1)
**BTK patent vessels**
0–1	15 (53.6)
2–3	13 (46.4)
BTK intervention	10 (35.7)
Stent fracture	2 (7.1)
ISCTO	25 (89.3)
Debulking devices	14 (50.0)
Silverhawk/Turbohawk	4 (14.3)
Angiojet	2 (7.1)
Rotarex	8 (28.6)
Bailout stent	1 (3.6)
In hospital complication	3 (10.7)

*Continuous data are presented as means ± standard deviation; categorical data are shown as n (%). ISCTO, in-stent chronic total occlusion*.

### Follow-Up Outcomes

**Figure 1:**
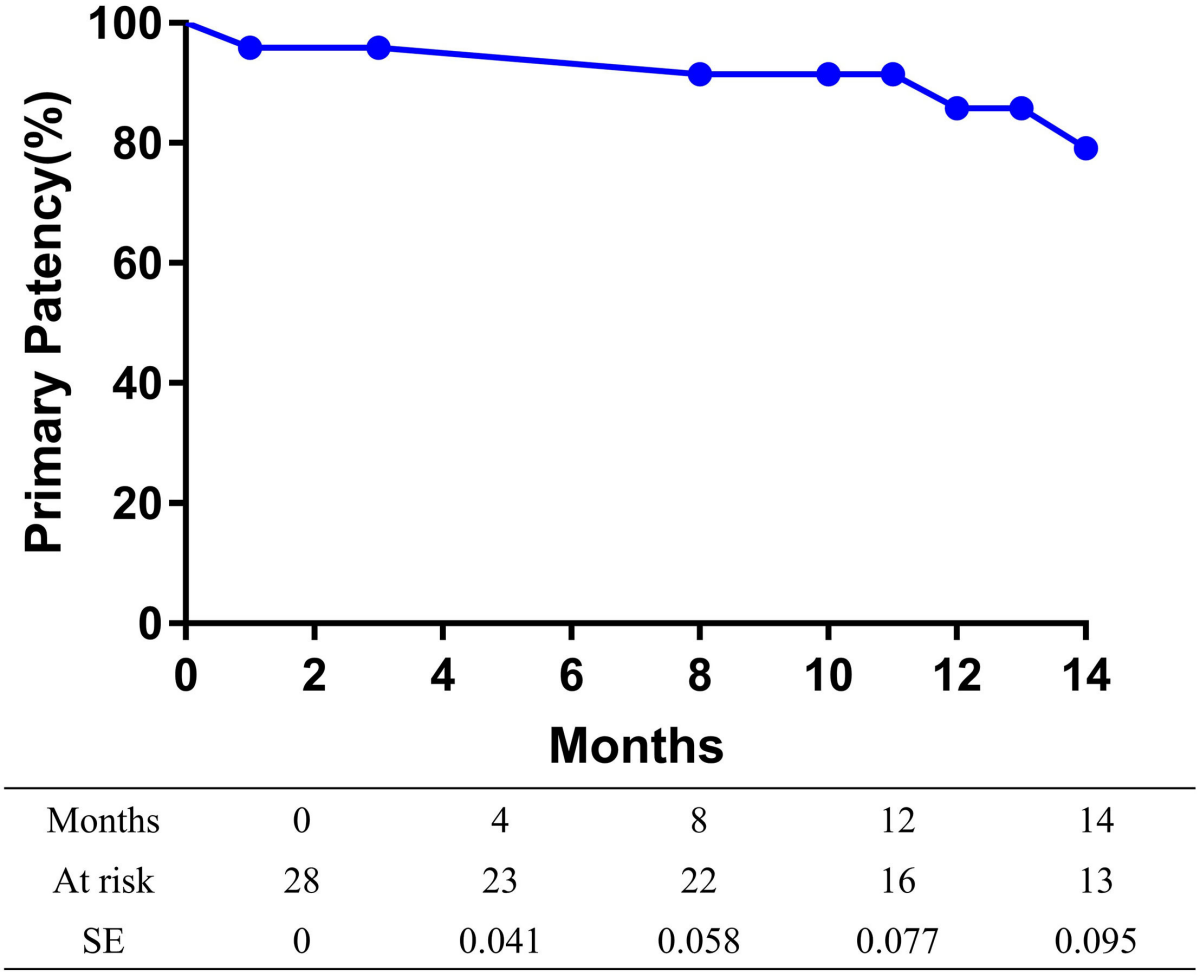
Kaplan-Meier curves of the primary patency of patients.

**Figure 2:**
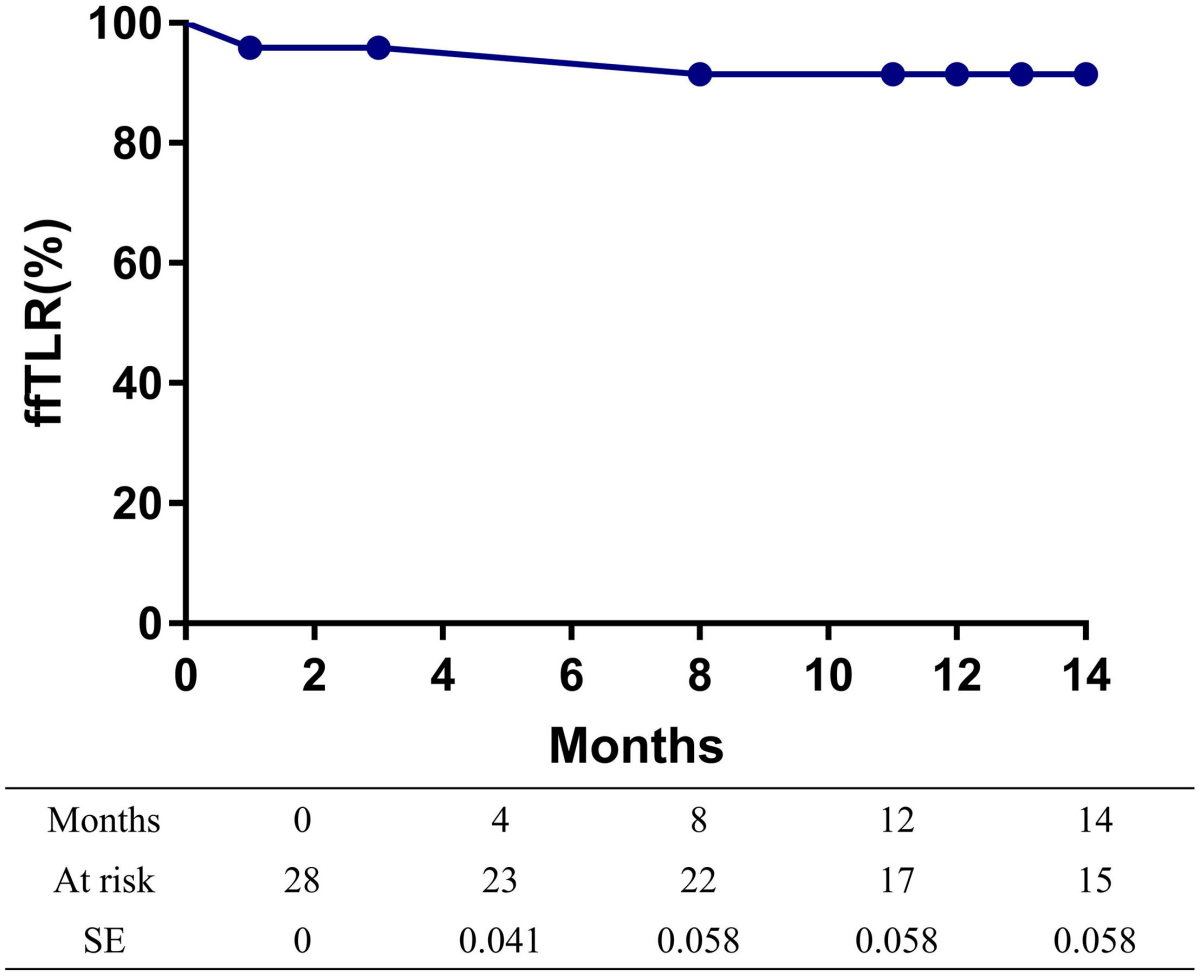
Kaplan-Meier curves of the freedom from target lesion revascularization (TLR) of patients.

The overall follow-up rate was 85.7% (24/28) and the mean follow-up time was 21.5 ± 10.3 months. Symptoms improved by at least 1 Rutherford category in 82.1% (23/28) of limbs at follow-up. The mean ABI value at follow-up was 1.05 ± 0.22, increasing significantly from baseline (*p* < 0.001). A total of two patients died at 8 and 29 months after the procedures, with heart attacks being the cause of death in both cases. The 14-month mortality rate was 7.1%. At 14 months after treatment, the primary patency rate and the freedom from TLR rate, as determined by the Kaplan-Meier estimates, were 79.2% (95% CI 60.6–97.8%; Figure 1) and 91.5% (95% CI 80.1–100%; Figure 2), respectively.

As shown in Table 3, the univariate analysis determined that the risk factor for restenosis was bailout stenting (*P* < 0.05). However, the Cox multivariate analysis showed that bailout stenting was not a risk factor for restenosis (*P* = 1.000, HR = 1.000, 95% CI 0–18,184.735). The univariate analysis also showed that diabetes and bailout stenting were possible risk factors for TLR (*P* < 0.05). However, the Cox multivariate analysis showed that neither was an independent risk factor for TLR.

**Table 3:** Univariate and multivariate analyses of the risk factors for 12-month primary patency and target lesion revascularization (TLR).

**Variables**	**Primary patency**			**TLR**		
	**Univariate**	**Multivariable**	**HR (95%CI)**	**Univariate**	**Multivariable**	**HR (95%CI)**
Age (≥65)	0.611			0.802		
Gender	0.581			0.301		
Hypertension	0.940			0.442		
Dyslipidemia	0.283			0.092		
Diabetes	0.490			0.042	1.000	1.000 (0.049–20.591)
Stroke	0.340			0.074		
Renal function Impairment	0.388			0.516		
Smoking	0.134			0.106		
Coronary artery disease	0.409			0.550		
Lesion length(≥150 vs. <150 mm)	0.342			0.442		
Ca+	0.340			0.902		
Severe Ca+	0.383			0.802		
Pre procedure outflow (0,1 vs. 2,3)	0.177			0.123		
Post procedure outflow (0,1 vs. 2,3)	0.515			0.279		
BTK intervention	0.635			0.301		
Ipsilateral iliac artery lesion	0.518			0.662		
Flow limiting dissection	0.623			0.763		
Bailout stent(yes vs. no)	<0.001	1.000	1.000 (0–18,184.735)	<0.001	1.000	1.000 (0–22,731.773)
Debulking devices(yes vs. no)	0.449			0.902		
Dissection (yes vs. no)	0.476			0.550		

## Discussion

In this single-center study, 28 patients with Tosaka class III femoropopliteal lesions were treated with DCBs and followed up for 14 months. The data showed that DCB treatment is safe in Tosaka class III patients, and the results were promising in terms of patency rates, freedom from TLR, and improved clinical symptoms.

The use of DCBs in treating femoral ISR lesions is expected to be superior to POBA due to the anti-hyperplasia properties of paclitaxel (24). In a prospective multicenter randomized trial, the authors claimed that treating superficial femoral artery ISR lesions with DCBs was associated with less recurrent restenosis and a better clinical outcome than POBA after a 12-month follow-up (18). In the PACUBA trial, DCBs showed a superior 12-month primary patency compared to POBA in lesions with an average length >17 cm (40.7 vs. 13.4%) (21). Another report has demonstrated that at the 24-month follow-up, DCBs were associated with a significant reduction of TLR compared to POBA (23). However, long-term results showed that at 3 years, the freedom from TLR rates was similar between the DCB and POBA groups (20). Considering the late catch-up phenomenon of drug-coated devices (17, 25), the long-term efficacy of DCBs in treating ISR lesions needs to be further investigated.

Tosaka et al. demonstrated that restenotic patterns were important predictors of recurrent ISR (8). They reported that the rate of recurrent ISR at 2 years after POBA was 84.8% in class III patients compared with 49.9% in class I patients and 53.3% in class II patients. Similar results were found in ISR lesions treated by DCBs. Recent research reported that the treatment of complex ISR lesions (classes II and III) was associated with an increased rate of recurrent restenosis compared with class I lesions (33.3% and 36.3 vs. 12.5%) (17). In the DEBATE-ISR study, the presence of a Tosaka class III occlusion was associated with a worse 3-year outcome in both the DCB and the angioplasty groups (20). However, contrasting results were reported by other researchers. In the PLAISIR Trial, no significant differences between Tosaka class I (25 lesions) and class II lesions (29 lesions) were noted in terms of freedom from TLR at 1 year after DCB treatment (22). According to the 1-year results of the PACUBA Trial, there was no significant difference between stenosis (Tosaka class I or II) and occlusion (Tosaka class III) in terms of primary patency and freedom from TLR (21). In our study, the 14-month primary patency was close to 80%, and the freedom from TLR rate was 91.5%. These results are promising, and DCBs should be considered the first-line treatment for femoropopliteal lesions.

Removing the neointimal hyperplasia and extra-cellular matrix of ISR with debulking devices is theoretically an attractive option. In a single-center randomized study, the combination of laser atherectomy and DCB in treating Tosaka class III ISR lesions was correlated with improved 12-month primary patency and freedom from TLR rates in comparison with DCB alone (26). Similarly, the combination of directional atherectomy with DCB was associated with greater freedom from TLR rate than POBA after directional atherectomy (27). In our study, 50% of lesions were treated with a combination of debulking and DCB. The use of debulking devices may help to improve clinical outcomes.

There are potential limitations in the present study. First, this was a nonrandomized and noncomparative study, and it was therefore not possible to compare DCBs and other treatment options in managing Tosaka class III lesions. Second, it was a single-center study, and bias related to the sample size and loss to follow-up may limit the results' generalizability. Third, the multivariable analyses could not be performed due to the small sample size. Further studies with larger sample sizes are warranted, and the risk factors of restenosis should be evaluated.

In conclusion, DCB angioplasty was shown to be a safe and effective treatment option in patients for Tosaka class III lesions at 14 months follow-up.

## Data Availability Statement

The raw data supporting the conclusions of this article will be made available by the authors, without undue reservation.

## Ethics Statement

The studies involving human participants were reviewed and approved by Human Investigations Committee of Peking University First Hospital. Written informed consent for participation was not required for this study in accordance with the national legislation and the institutional requirements.

## Author Contributions

All authors listed have made a substantial, direct and intellectual contribution to the work, and approved it for publication.

## Conflict of Interest

The authors declare that the research was conducted in the absence of any commercial or financial relationships that could be construed as a potential conflict of interest.
